# Supplementary material for: Interphotoreceptor matrix proteoglycans IMPG1 and IMPG2 proteolyze in the SEA domain and reveal localization mutual dependency
Source: Sci Rep. 2022 Sep 15;12:15535. doi: 10.1038/s41598-022-19910-1 (PMC9478142; doi:10.1038/s41598-022-19910-1)
Supplement: Supplementary file 1 — Supplementary Information. [file 41598_2022_19910_MOESM1_ESM.docx]

**Supplementary material**

**Interphotoreceptor matrix proteoglycans IMPG1 and IMPG2 proteolyze in the SEA domain and reveal localization mutual dependency**

Benjamin Mitchell, Chloe Coulter, Werner J. Geldenhuys, Scott Rhodes, Ezequiel M Salido

Supplementary Figure S1

Supplementary Figure S2

Supplementary Figure S3


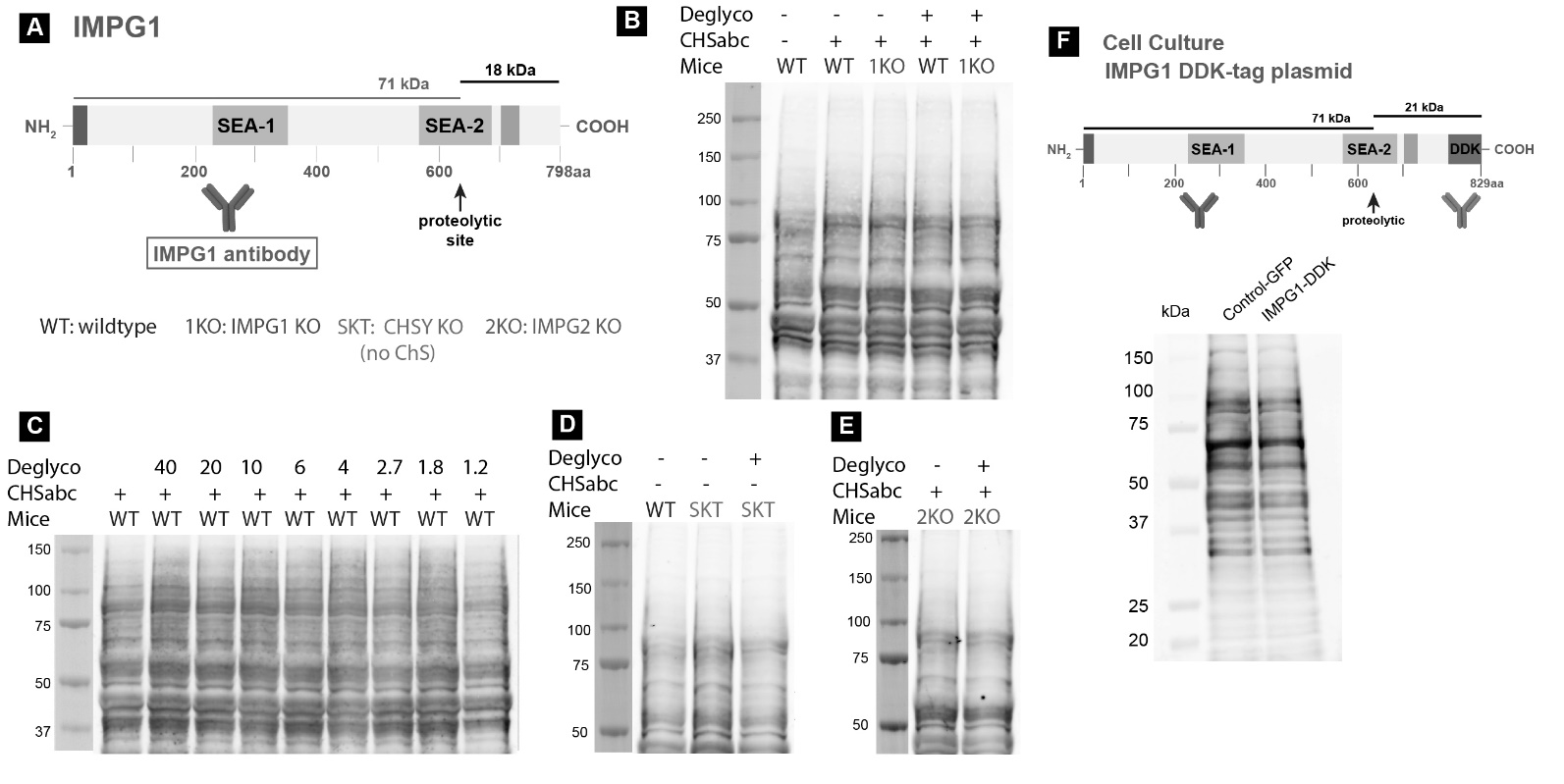


**Supplementary Figure S1**. Western blot loading control of Figure 2 by total protein staining assay.


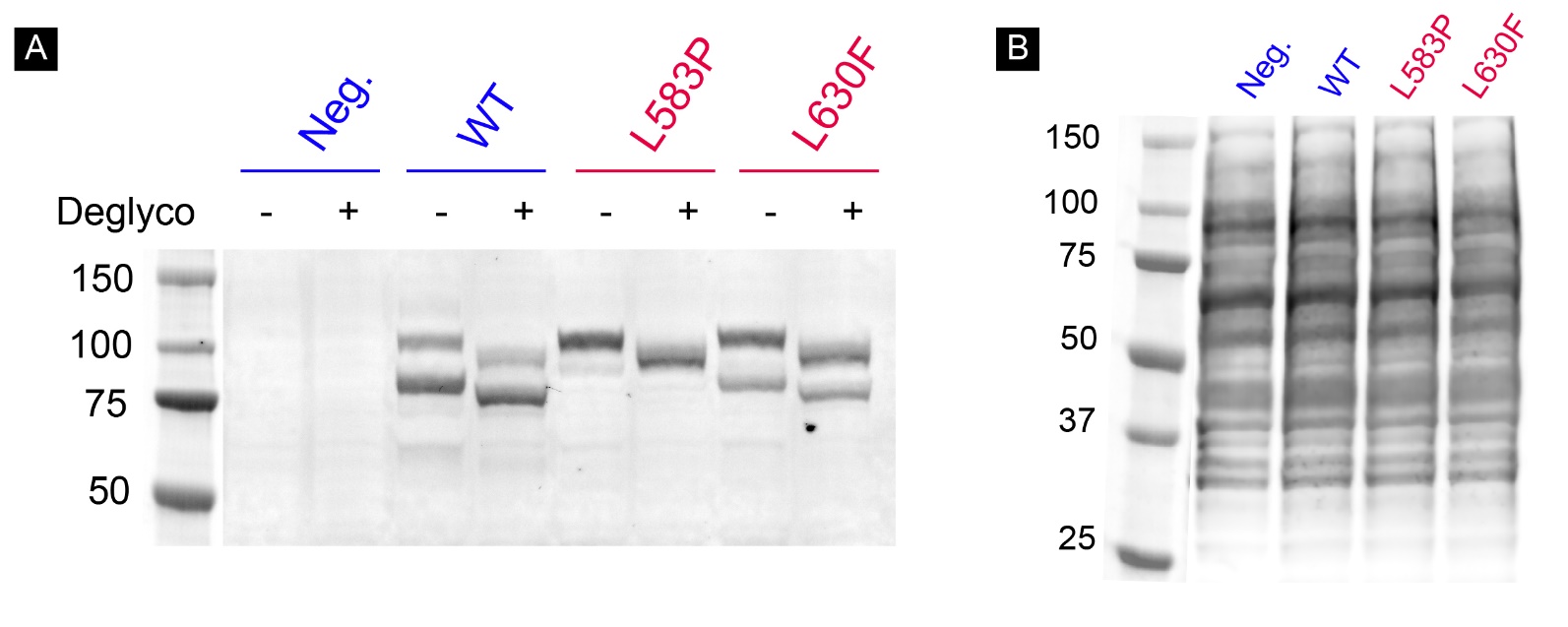


**Supplementary Figure S2**. **HEK293 cells glycosylate IMPG1 unrelatedly to protein proteolysis.** **A**, the same samples used in figure 3, IMPG1 WT and mutated proteins expressed in HEK293 cells treated with or without deglycosylation protein mix (Deglyco). Glycosylation in IMPG1 generates a ~5kDa increase in the relative molecular mobility in proteolyzed and non-proteolyzed molecules. **B**, Western blot loading control of Figure 3 by total protein staining assay. The experiment was reproduced 3 times with different samples.


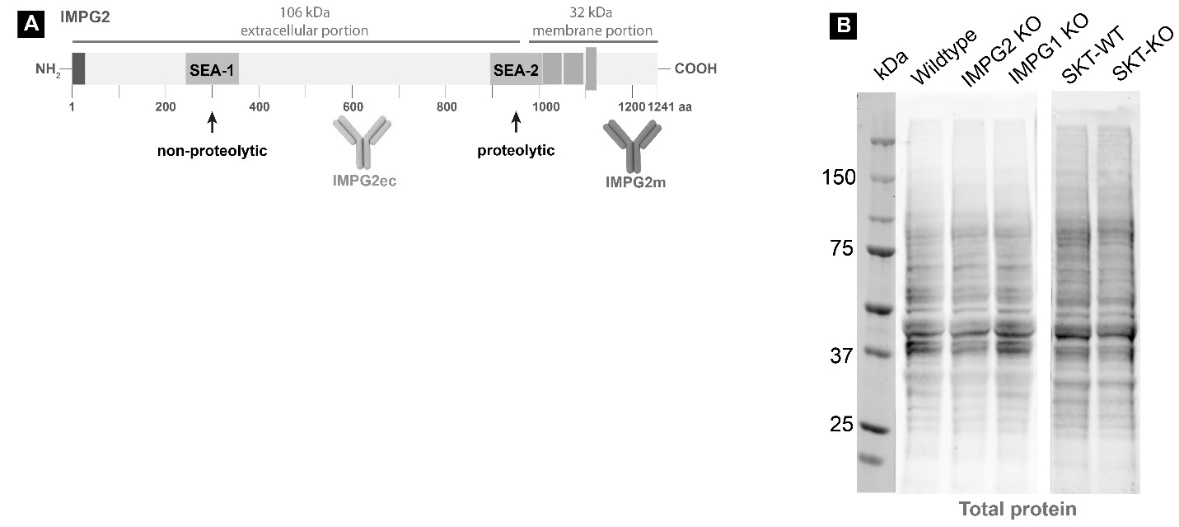


**Supplementary Figure S3**. Western blot loading control of Figure 4b in the text of the manuscript by total protein staining assay.
